# Perfusion process with tangential flow filtration for oncolytic VSV-GP production

**DOI:** 10.3389/fbioe.2025.1588293

**Published:** 2025-05-30

**Authors:** Orsolya Hamusics, Anja Wittmann, Katrin Hasler, Sabrina Müller, Daniel Birk, Ingo H. Gorr, Alexander Brix, Jorge Soza-Ried

**Affiliations:** ^1^ Viral Therapeutics Center, Boehringer Ingelheim Pharma GmbH & Co. KG, Biberach an der Riss, Germany; ^2^ Global Innovation and Alliance Management, Boehringer Ingelheim Pharma GmbH & Co. KG, Biberach an der Riss, Germany

**Keywords:** vesicular stomatitis virus, oncolytic virus, perfusion, tangential flow filtration, design of experiments

## Abstract

The oncolytic vesicular stomatitis (VSV)-GP virus is a promising therapeutic against cancer. To ensure clinical efficacy, doses with high titers are required, which poses a challenge for the manufacturing process. Perfusion cultivation processes with high cell densities have attracted great interest to improve the production titer. This work aimed to enhance the titer of the VSV-GP production process with suspension human embryonic kidney 293 (HEK293) cells by using perfusion with tangential flow filtration (TFF) and virus retention. For this purpose, six potential critical process parameters were evaluated using I-optimal design of experiments (DoE). The study showed that several input parameters and their interactions have significant impact on the infectious titer. Increasing the seeding cell density significantly improved the infectious titer, allowing infection at up to 46.6 × 10^6^ cells mL^-1^ without decrease in the cell-specific virus yield. Keeping the perfusion pause after infection at minimum (1.1–1.3 h) and subsequently start the perfusion with a higher exchange rate (0.045–0.051 nL cell^-1^ d^-1^) was shown to be beneficial. The process was sensitive to shear stress and thus, the optimal crossflow rate was between 44 and 55 mL min^-1^, which corresponds to 950–1150 s^-1^ shear rate. By optimizing the perfusion process, the titer reached up to 5.1 × 10^10^ TCID_50_ mL^-1^, which is 17-fold higher than in batch cultivation. Overall, this work presents perfusion cultivation as an efficient technology to improve the VSV-GP titer with virus retention.

## Introduction

Oncolytic virotherapy is an emerging therapeutic approach, which uses oncolytic viruses to selectively destroy malignant tissues (Shalhout et al., 2023). Vesicular stomatitis virus (VSV) is a promising candidate for oncolytic virotherapy. It has a broad cell tropism and a fast replication cycle, which ends in high titer (Hastie et al., 2013). VSV also has a capacity to carry transgenes such as tumor antigens or immunostimulatory molecules to enhance the therapeutic effect (Kimpel et al., 2018). Human infection with VSV is rare and only causes mild flu-like symptoms (Hastie et al., 2013). The tumor selectivity of the virus is based on the frequently aberrated type I interferon response in cancer cells that reduces the antiviral defense (Kimpel et al., 2018). The chimeric vesicular stomatitis virus, VSV-GP was created by substituting the envelope glycoprotein (G) of the VSV with the glycoprotein (GP) of the lymphocytic choriomeningitis virus (LCMV). Due to this modification, VSV-GP lacks VSV’s inherent neurotoxicity and is able to escape the humoral immune response (Muik et al., 2014). VSV-GP has shown potential to treat, e.g., glioblastoma (Muik et al., 2014), melanoma (Kimpel et al., 2018) and ovarian cancer (Dold et al., 2016).

VSV production processes have been described with adherent cell cultures on diverse growth matrices such as macroporous gelatin carriers (Lim et al., 1999), microcarriers (Kiesslich et al., 2020), Fibra-Cel disks (Rosen et al., 2022) and in Scale-X™ hydro fixed-bed bioreactor (Kiesslich et al., 2020). A GMP-conform production process in a 10-layer cell factory has also been reported (Ausubel et al., 2011). Due to the easier process scale-up and handling, adaptation of adherent cells to suspension growth has received a high interest. Accordingly, several recently published VSV production processes have utilized suspension cells (Paillet et al., 2009; Elahi et al., 2019; Gélinas et al., 2019; Kiesslich et al., 2021; Gautam et al., 2022; Göbel et al., 2022).

Perfusion is an advanced cultivation technique that enables continuous culture medium exchange while cells are retained in the bioreactor. Consequently, higher cell densities and higher titers can be achieved with perfusion compared to a classical batch cultivation process (Bock et al., 2011; Gálvez et al., 2012). Perfusion processes also have the potential to achieve higher volumetric productivities and operate with a smaller footprint compared to fed-batch processes due to the higher cell densities (Pollock et al., 2013; Tapia et al., 2016; Kiesslich et al., 2021). Hollow fiber modules with tangential flow filtration (TFF) or alternating tangential flow filtration (ATF) are widely used to retain cells in the bioreactor (Nikolay et al., 2018; Vázquez-Ramírez et al., 2018; Gränicher et al., 2019; Lavado-García et al., 2020). They are easy to scale up based on the surface area of the hollow fiber filter (Ozturk and Kompala, 2005). Previously, the TFF perfusion with a peristaltic pump was linked to higher shear stress and filter fouling compared to the ATF system (Schwarz et al., 2020). Nevertheless, exchanging the peristaltic pump to a low shear centrifugal pump presents new opportunities for the TFF technology (Wang et al., 2017). To achieve high virus titers, virus production conditions need careful optimization (Gálvez et al., 2012; Tapia et al., 2016; Elahi et al., 2019; Wu et al., 2021). Implementing an appropriate feeding or medium exchange strategy is crucial to promote high cell-specific virus yields at high cell densities (Lindsay and Betenbaugh, 1992; Maranga et al., 2003; Kamen and Henry, 2004).

Ambr^®^ 250 High Throughput bioreactor systems (Sartorius Stedim Biotech) have gained popularity for accelerating process development using 12 or 24 disposable mini bioreactors (Tai et al., 2015; Joe et al., 2021). The reliability of the bioreactors as scale-down models has been demonstrated in several studies (Xu et al., 2017; Manahan et al., 2019; Zhang et al., 2019). One of the key features of the Ambr^®^ 250 system is the individual automatic control of cultivation parameters, including dissolved oxygen (DO), pH, foam level, bolus addition and feeding. The automated sampling and sample analysis provide real-time data for controlling loops and substantially decrease manual work. These features promote the use of design of experiments (DoE) to analyze the impact of variables and their interactions on the process performance (Tai et al., 2015). Additionally, the high automatization minimizes human error and enables more consistent processes.

In this study, we aimed to improve the titer of the oncolytic VSV-GP production process with suspension HEK293 cells using TFF perfusion and Ambr^®^250 High Throughput bioreactors. Firstly, different medium exchange rates were investigated for cultivating suspension HEK293 cells in perfusion. Subsequently, a DoE approach was applied to identify process parameters with potential impact on the virus production, including the seeding cell density, time of infection (TOI), multiplicity of infection (MOI), perfusion pause after infection as well as the medium exchange rate and crossflow rate post infection. Finally, the perfusion process was optimized and compared to the batch production process in terms of titer and productivity.

## Materials and methods

### Cell and virus

HEK293-F cells (#11625019, Thermo Fisher Scientific, Waltham, MA, United States), hereafter referred to as HEK293 cells, were cultivated in a serum-free proprietary medium with 4 mM GlutaMAX™ (#35050061, Gibco, Thermo Fisher Scientific, Waltham, MA, United States) at 37°C, 5% CO_2_ and 120 rpm with 25 mm orbit shaker in non-baffled shaking flasks. Cells were passaged every 3 or 4 days with 2.5 × 10^5^ or 4 × 10^5^ cells mL^-1^ seeding density, respectively.

For infections, a recombinant VSV-GP virus vector stock (6.0 × 10^8^ TCID_50_ mL^-1^) was used in which the glycoprotein (G) of the VSV was replaced by the glycoprotein (GP) of LCMV as described before (Muik et al., 2014). The virus seed stock had a titer of 6.0 × 10^8^ TCID_50_ mL^-1^.

### Batch cultivation

Batch cultivations were performed in 250 mL Ambr^®^ 250 mammalian bioreactors (#001-5G25, Sartorius, Göttingen, Germany). Cells were seeded at 4.0 × 10^5^ cells mL^-1^ in 200 mL working volume. The pH was set to 7.2 with a negative dead band of 0.2 and controlled with CO_2_ as well as 1 M Na_2_CO_3_. The cultivation temperature was 37°C. Cells were agitated at 492 rpm with two pitched blade impellers (d = 26 mm). The DO was maintained at 50% with a mixture of air and oxygen. Initially, only air was sparged up to 1.75 mL min^-1^. Then, oxygen was added up to 80 mL min^-1^ to keep the DO set point. Cultures were infected with an MOI of 0.0004 after 56 h of cultivation. The cultivation temperature was shifted from 37°C to 34°C directly before infection.

### Perfusion cultivation without infection

Perfusion cultivations were conducted in 250 mL Ambr^®^ 250 bioreactors with TFF and 30 kDa modified polyether sulfone (mPES) filter (#001-5G83, Sartorius, Göttingen, Germany). The TFF filter had 75 cm^2^ filter area with 20 cm effective length, 20 fiber count and 1 mm lumen bore. Cells were seeded at 4.0 × 10^5^ cells mL^-1^ in 220 mL working volume. Cells were cultivated with the same pH, temperature, agitation and DO set points and control strategy as described by the batch cultivations. To eliminate present foam, 100 µL antifoam (FoamAway™, #A1036902, Thermo Fisher Scientific, Waltham, MA, United States) was added to the cultures.

Perfusion was started 16 h after seeding using the growth medium with increased, 10 g L^-1^ glucose content. The cell-specific perfusion rate (CSPR) was either 0.029, 0.051 or 0.073 nL cell^-1^ d^-1^. The minimum value of the medium exchange rate was set to 0.3 reactor volume per day (RV d^-1^). If the glucose concentration fell below 5 g L^-1^ during the perfusion, a glucose bolus was added up to twice a day from a 500 g L^-1^ stock solution to reach 5 g L^-1^. The crossflow rate was 40 mL min^-1^.

### DoE for the virus production

Six input parameters were varied at three levels to optimize the virus production in TFF perfusion vessels with 30 kDa mPES filter (#001-5G83, Sartorius, Göttingen, Germany). An I-optimal DoE was employed to develop a response surface model that considered interactions and quadratic effects of the parameters (Table 1). To test different cell densities for infection, the seeding viable cell density (VCD) (1 × 10^6^, 2.5 × 10^6^ or 4 × 10^6^ cells mL^-1^) and the TOI (74, 98 or 122 h) were varied. Seed cultures were concentrated by centrifugation (180 g, 5 min, RT) prior to inoculation to ensure that a maximum split ratio of 20% (ratio of spent medium to fresh medium) was not exceeded. Perfusion was started 16 h after seeding with 0.051 nL cell^-1^ d^-1^ CSPR with a minimum medium exchange rate of 0.3 RV d^-1^. The same glucose strategy was adopted as in the perfusion cultivations without infection. The crossflow rate was set to 40 mL min^-1^. Before infection, the cultivation temperature was shifted from 37°C to 34°C. Then, the perfusion was halted by stopping the inflow and outflow of the medium as well as the crossflow within the system. Following this, cells were infected with an MOI of 0.04, 0.004 or 0.0004. Perfusion resumed 1, 8 or 16 h after infection with varying CSPRs (0.015, 0.033 and 0.051 nL cell^-1^ d^-1^) and crossflow rates (25, 40 and 70 mL min^-1^). Virus samples were collected at 22, 36, 40 and 46 h post infection (hpi).

**TABLE 1:** DoE input parameters and their levels. Samples were collected at 22, 36, 40 and 46 hpi.

Input parameter	Level −1	Level 0	Level +1
Seeding VCD [x 10^6^ cells mL^-1^]	1.0	2.5	4.0
TOI [h]	74	98	122
log_10_ MOI	−3.4	−2.4	−1.4
CSPR after TOI [nL cell^-1^ d^-1^]	0.015	0.033	0.051
Perfusion pause after TOI [h]	1	8	16
Crossflow rate after TOI [mL min^-1^]	25	40	70

30 experiments were planned in three blocks within the DoE. Additionally, three perfusion runs in block 3 and 12 runs in block 4 were performed to improve the quality and prediction of the statistical model. Up to 12 experiments were conducted within a single block. Input parameter settings of the perfusion cultivations are provided in the attachment (Supplementary Table S1).

### Statistical analysis

The analysis and calculations for the DoE were performed with Design-Expert 13.0.5.0 software (Stat-Ease, RRID:SCR_022671).

A split plot design was used in the analysis to incorporate the dependency structure of the data, considering each DoE parameter as a hard-to-change factor and hpi as an easy-to-change factor. Therefore, additionally to the response surface model of the six DoE parameters, the linear and quadratic effect of hpi as well as their interactions with the DoE factors were considered.

Models were fitted using restricted maximum likelihood (REML) analysis and selected via stepwise backward selection with the Akaike information criterion corrected for small sample size (AICc) as implemented in Design-Expert. Model diagnostics were performed by visual inspection of residual normality and dispersion. Outlier detection was performed via difference in fits (DFFITs) and studentized residuals plots. Due to the presence of heteroscedasticity in the data, a square root transformation was applied for the analysis.

### Analytical methods

#### Cell counting

Ambr^®^ 250 bioreactor cultures were automatically counted with the integrated BioProfile^®^ FLEX2 analyzer (Nova Biomedical, Waltham, MA, United States) to enable the automatic adjustment of the perfusion rate. Additionally, to account for cell aggregation during the cultivation process (Supplementary Figure S1), cells were also counted offline with the Nucleocounter^®^ NC-202™ (Chemometec, Allerod, Denmark). When the aggregation level reached 20%, samples were treated with Lysis 1 buffer (#910–0010, Chemometec, Allerod, Denmark) to disaggregate cells before counting. A conversion factor of 1.36 was considered between the BioProfile^®^ FLEX2 and Nucleocounter^®^ NC-202™ for the automatic perfusion rate adjustment to account for method differences.

#### Metabolites

Glucose and lactate were automatically measured with the BioProfile^®^ FLEX2 analyzer.

#### Virus titer

To harvest the virus from the cell-containing suspension, samples were incubated with sodium chloride as described previously (Gautam et al., 2022). Afterwards, samples were centrifuged (1000 g, 5 min, RT), and the supernatant was aliquoted and stored at −80°C until analysis. Infectious virus titer was determined with the automated, label-free tissue culture infectious dose 50 (TCID_50_) assay as described previously (Hochdorfer et al., 2022). The geometrical mean of the titer was calculated from single measurements on three different days.

### Calculations

#### Cell-specific virus yield

Cell-specific virus yield (CSVY, [TCID_50_ cell^-1^]) was calculated according to Equation (1) to account for the cell growth after infection. Accordingly, CSVY was determined by dividing the maximum observed infectious titer (Vir_max_, [TCID_50_ mL^-1^]) by the maximum VCD (VCD_max_, [cells mL^-1^]) observed at TOI or thereafter (Coronel et al., 2019; Gränicher et al., 2019).

$$CSVY=\frac{{Vir}_{\max}}{{VCD}_{\max}}$$ (1)

#### Space-time yield

Space-time yield (STY, [TCID_50_ mL^-1^ d^-1^]) was determined based on the maximum infectious titer and process time (t, [d]) as shown in Equation 2:

$$STY=\frac{{Vir}_{\max}}{t}$$ (2)

#### Volumetric productivity

The volumetric productivity (P_V_, [TCID_50_ mL^-1^ d^-1^]) was calculated according to Equation 3 based on the maximum virus titer, the working volume of the bioreactor (V_W_, [mL]) as well as the consumed medium (V_Medium_, [mL]) and process time until Vir_max_ was reached:

$$P_{V}=\frac{{Vir}_{\max}\times V_{W}}{V_{Medium}\times t}$$ (3)

## Results

### Cell growth with perfusion cultivation

**FIGURE 1:**
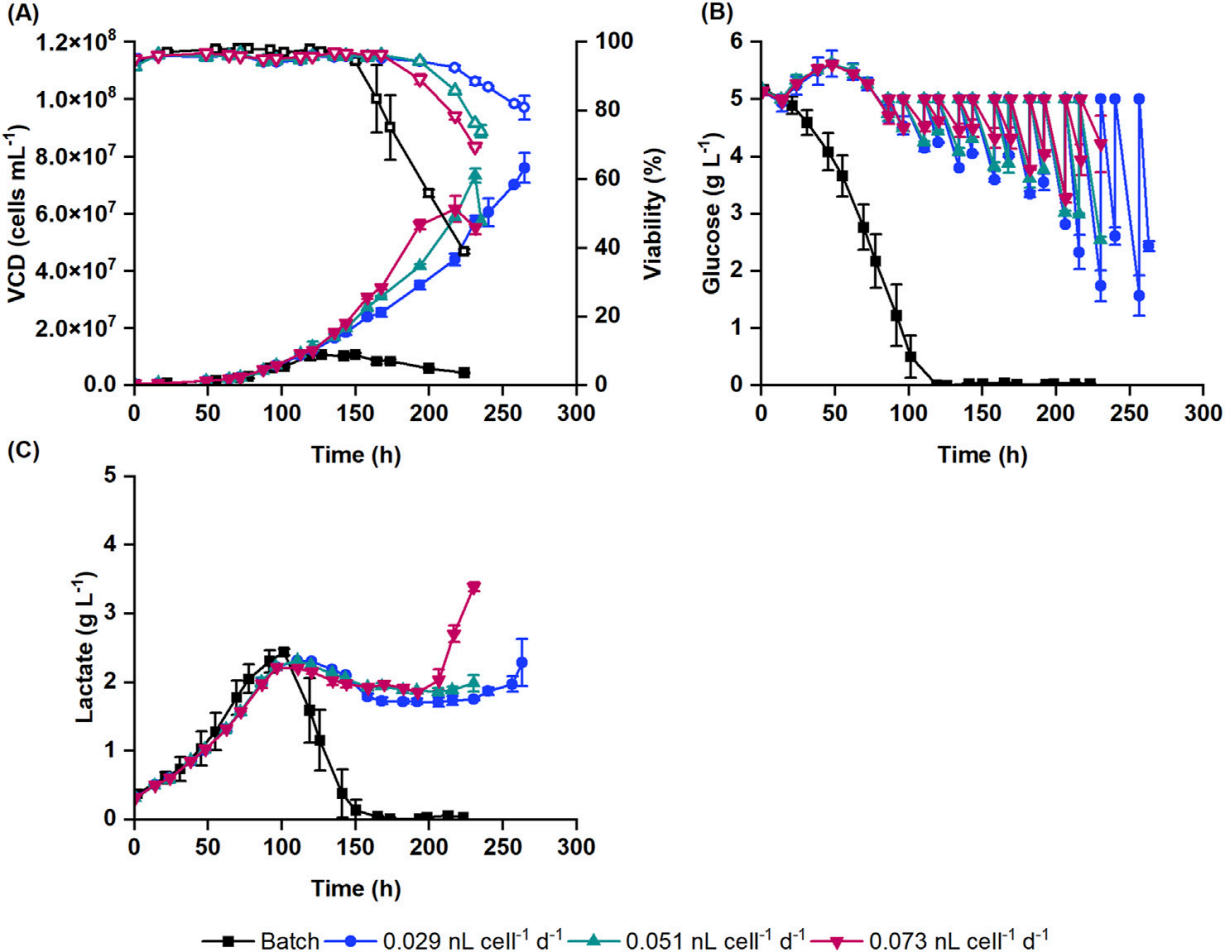
Suspension HEK293 cell cultivation in batch and perfusion cultivation mode with 0.029, 0.051 and 0.073 nL cell^-1^ d^-1^ cell-specific perfusion rates (CSPR). **(A)** Viable cell density (VCD) (filled symbols) and viability (empty symbols) obtained with Nucleocounter^®^ NC-202™ are shown along with the **(B)** glucose and **(C)** lactate concentrations. Data represent the mean and standard deviation of biological duplicates.

Perfusion cultivation supports cell growth to high cell densities. To test the impact of medium exchange on the suspension HEK293 cell growth, processes with different CSPRs (0.029, 0.051 and 0.073 nL cell^-1^ d^-1^) were tested and compared to the reference batch cultivation process (Figure 1).

Batch cultures reached a concentration of 10.5 ± 1.6 × 10^6^ cells mL^-1^ at 119 h. In comparison, cells with the lowest perfusion rate (0.029 nL cell^-1^ d^-1^) reached a 6-fold higher VCD of 76.0 ± 5.2 × 10^6^ cells mL^-1^. Similar VCD levels were reached at an earlier timepoint with 0.051 nL cell^-1^ d^-1^ perfusion rate (73.3 ± 2.4 × 10^6^ cells mL^-1^). Cultures with 0.073 nL cell^-1^ d^-1^ medium exchange rate had a slightly lower final cell concentration (61.7 ± 4.6 × 10^6^ cells mL^-1^) compared to other perfusion cultures (Figure 1A). This was accompanied by a faster increase in transmembrane pressure, which indicates filter clogging and decreased filtration efficiency. Overall, the growth rate of perfusion cultures slightly increased with the perfusion rate. The specific growth rates were 0.022, 0.025 and 0.027 h^-1^ at perfusion rates of 0.029, 0.051 and 0.073 nL cell^-1^ d^-1^, respectively. The viability of the perfusion cultures dropped below 90% under all conditions usually when the cell concentration reached between 56.1 × 10^6^ and 59.0 × 10^6^ cells mL^-1^ (Figure 1A). To cover the increasing oxygen demand of the cultures, the oxygen flow rate was continuously increased during the cultivation and reached 0.22–0.31 vessel volumes per minute (VVM) when the viability dropped below 90%. Consequently, this resulted in increased shear forces in the bioreactor due to bubble bursting, potentially causing higher cell death at higher cell densities. Additionally, bubbles trapped within the membrane may have reduced filtration efficiency, contributing to the decline in cell viability.

In batch cultures, glucose was completely depleted by 119 h, marking the end of the exponential cell growth phase (Figure 1B). Following this, cells began to consume lactate. As the lactate was depleted (from 150 h onwards), the cell density and viability started to decline (Figure 1C). In perfusion cultures, glucose was continuously supplied alongside the medium exchange, starting at 16 h post seeding. Initially, this led to a small increase in the glucose concentration. From 86 h onwards, as the glucose concentration dropped below 5 g L^-1^, a glucose bolus was applied to counterbalance the consumption of cells (Figure 1B). Cells produced lactate up to 2.2–2.3 g L^-1^. After 110 h, the lactate concentration gradually decreased to 1.7–1.9 g L^-1^ as the cells metabolized the lactate. Once the viability of the cells began to decline, the lactate concentration increased again in the bioreactor (Figure 1C).

For the subsequent DoE study, 0.051 nL cell^-1^ d^-1^ perfusion rate was selected, which promoted a higher specific growth rate than the 0.029 nL cell^-1^ d^-1^ perfusion rate, while also keeping the medium consumption within reasonable limits.

### DoE and statistical model of the virus production

Process parameters with potential influence on the virus titer were evaluated in perfusion cultures with an I-optimal DoE. The seeding VCD (1 × 10^6^, 2.5 × 10^6^ and 4 × 10^6^ cells mL^-1^) and TOI (74, 98 and 122 h) were varied to test different cell densities and timepoints for the infection. These levels were chosen to cover cell densities up to a maximum of 59.0 × 10^6^ cells mL^-1^, where the culture’s viability begins to decline (Figure 1A). To ensure an efficient infection, the impact of MOI (0.0004, 0.004, 0.04) was also evaluated. The selection of MOI levels was based on the current MOI of 0.0004 in the reference batch process, which was set as a minimum threshold to maintain practical handling in large-scale production. A short perfusion pause of 1–15 h is frequently used after infection to facilitate virus entry into cells (Yuk et al., 2004; Gálvez et al., 2012; Genzel et al., 2014) and in certain instances, first virus replication cycles (Luitjens and Herk, 2011). Therefore, the study included perfusion pauses of 1, 8 and 16 h. Once the perfusion started again, the necessity of the medium exchange was tested with different CSPRs (0.015, 0.033 and 0.051 nL cell^-1^ d^-1^). The levels of CSPR were selected to balance between conditions known to support high viability and high cell density cultures (0.033 and 0.051 nL cell^-1^ d^-1^) (Figure 1A) and conditions (as low as 0.015 nL cell^-1^ d^-1^) to keep the medium consumption within an acceptable range. The shear-sensitivity of the virus and/or cells was tested by applying different crossflow rates (25, 40 and 70 mL min^-1^) that were either lower or higher than during the cell cultivation prior to infection and covered shear rates from 400 s^-1^ to 1500 s^-1^. The crossflow rate levels were chosen to fall within the recommended operational range of Ambr^®^ 250 system specified by the manufacturer. In the reference batch process, the virus production kinetics reaches plateau within 46 hpi. Therefore, we expected to reach the maximum virus titer in perfusion by 46 hpi as well and collected virus samples at 22, 36, 40 and 46 hpi. 30 perfusion cultivations were planned for the DoE study and further 15 runs were added to improve the model quality and prediction (Supplementary Table S1).

**FIGURE 2:**
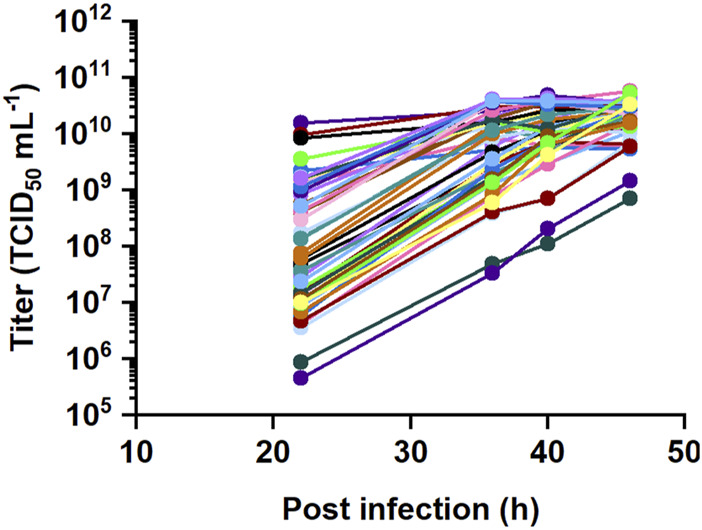
DoE study for recombinant VSV-GP production with 45 perfusion runs. Six input parameters were varied at three levels to maximize the virus titer, including seeding VCD, TOI, MOI as well as CSPR, perfusion pause and crossflow rate after infection.

The titers obtained in the DoE study varied in the range of two log_10_ steps between 7.1 × 10^8^ and 5.6 × 10^10^ TCID_50_ mL^-1^ at 46 hpi (Figure 2). Raw data was fitted using REML, incorporating time as an easy-to-change factor to assess the time-dependency of the data and considering each bioreactor as a group. The residual analysis showed heteroscedasticity and therefore, the data was square root transformed. The validity of the fitted models was assessed by model diagnostics. A residual analysis was performed to verify assumptions of normally distributed and homoscedastic data as well as to detect outliers. A visual inspection was performed to assess whether the residuals of the datapoints approximate a straight line in the normal quantile plot and are randomly scattered around the zero line in the residuals vs predicted plot.

The *R* ^2^ of the model equaled 0.869, i.e., the model describes 86.9% of the variance within the data. Significance of the input parameters were analyzed considering main effects and interactions. The analysis of variance (ANOVA) of the whole-plot as well as the subplot (effect of DoE factors on kinetics) showed a significant model with p-values <0.0001 (Table 2). The coefficient of variation (CV) of the model, calculated as the root mean square error divided by the mean, was 32%, aligning well with the expected method variability. The datapoints followed the diagonal line in the actual vs predicted plot.

**TABLE 2:** p-values and coefficient estimates of the statistical model.

Terms	p-value^a^	Coefficient estimate	Standard error
Whole-plot	<0.0001		
[Seeding VCD]	0.0003	9220.8	2492.0
[TOI]	0.0448	5670.2	2803.2
[log_10_ MOI]	<0.0001	24928.7	4160.1
[CSPR]	0.0466	8619.3	4296.4
[Perfusion pause]	<0.0001	−26389.6	4211.2
[Crossflow rate]	0.0711	5209.8	2866.5
[Seeding VCD] × [Crossflow rate]	0.0544	−5708.2	2944.4
[TOI] × [log_10_ MOI]	0.0742	5457.8	3036.2
[TOI] × [Perfusion pause]	0.0139	−7073.1	2843.7
[log_10_ MOI] × [CSPR]	0.0290	−6774.0	3072.8
[log_10_ MOI] × [Perfusion pause]	0.0033	8552.8	2860.7
[Perfusion pause]^2^	0.0411	12369.7	6006.4
[Crossflow rate]^2^	0.0377	−12055.4	5752.7
Subplot	<0.0001		
[hpi]	<0.0001	57922.8	2938.9
[Seeding VCD] × [hpi]	0.0009	9909.8	2935.6
[TOI] × [hpi]	0.0570	6123.2	3192.4
[log_10_ MOI] × [hpi]	0.0088	−8515.7	3210.7
[CSPR] × [hpi]	0.4339	2569.3	3274.6
[Perfusion pause] × [hpi]	0.0657	−5954.3	3212.0
[hpi]^2^	0.7563	1459.3	4694.4
[log_10_ MOI] × [hpi]^2^	0.0660	−9650.0	5212.2
[CSPR] × [hpi]^2^	0.0426	−11211.7	5482.1
[Perfusion pause] × [hpi]^2^	0.0009	18048.3	5348.8

^a^ p-values less than 0.0500 indicate that model terms are significant with 95% confidence. Values greater than 0.1000 indicate that model terms are not significant. Not significant model terms were included if they were present in higher order interactions.

**FIGURE 3:**
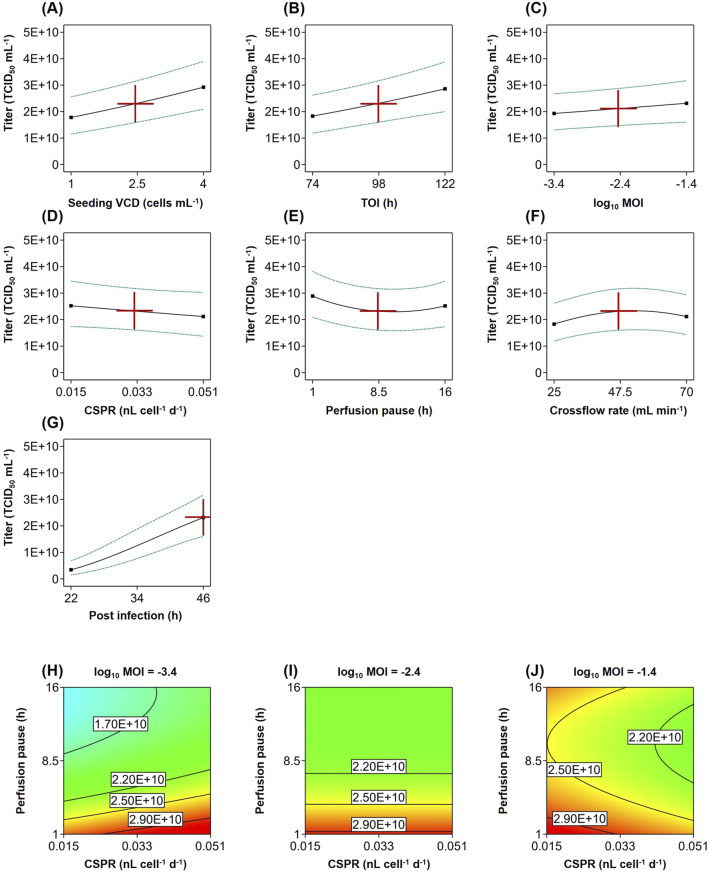
Statistical model of recombinant VSV-GP production in perfusion cultures. **(A–G)** Impact of input parameters on the infectious virus titer (solid line) with 95% confidence interval (dashed line) at center point condition at 46 hpi. **(H–J)** Interaction of MOI with the CSPR and perfusion pause after infection at three log_10_ MOI levels from −3.4 to −1.4 at center point condition at 46 hpi.

Increasing the seeding VCD or choosing a later timepoint for infection led to overall higher cell concentrations and significantly improved the titer (Figures 3A,B).

The MOI as well as its interaction with the CSPR and perfusion pause also had a significant effect on the titer (Table 2). While increasing the MOI generally had a positive impact (Figure 3C), the direction of the relationship with the titer depended on the parameter settings. For example, with 1 h perfusion pause and high 0.051 nL cell^-1^ d^-1^ perfusion rate, an MOI of 0.0004 (log_10_ MOI = −3.4) was more beneficial than a higher MOI of 0.04 (log_10_ MOI = −1.4) (Figures 3H,J). In processes with lower perfusion rate and longer perfusion pause, the titer increased with higher MOIs (Figures 3H–J).

Lowering the perfusion rate for the virus production generally decreased the titer (Figure 3D). Also, pausing the perfusion after the infection generally reduced the titer (Figure 3E). The interaction between the perfusion pause and the TOI suggests that pausing the perfusion had a greater impact on cultures, which were infected at later timepoints (Table 2).

The crossflow rate was only significant as a quadratic term, supporting higher titers in the middle of the tested range (Figure 3F).

Including the sampling time in the model enabled us to capture the virus production kinetics (Figure 3G) and its interaction with multiple input parameters. Applying a higher seeding cell density, higher MOI and higher cell-specific perfusion rate led to a faster increase in titer, whereas using a longer perfusion pause after infection hindered the virus production (Table 2).

Overall, all tested input parameters exhibited a substantial impact on the titer. The model analysis revealed multiple significant interactions between parameters as well as between parameters and time, underscoring the intricate relationships within the biological system. The fitted model showed a good quality, indicating that it can be used to optimize the input parameters to achieve high infectious titer.

### Optimization of virus production with perfusion

**FIGURE 4:**
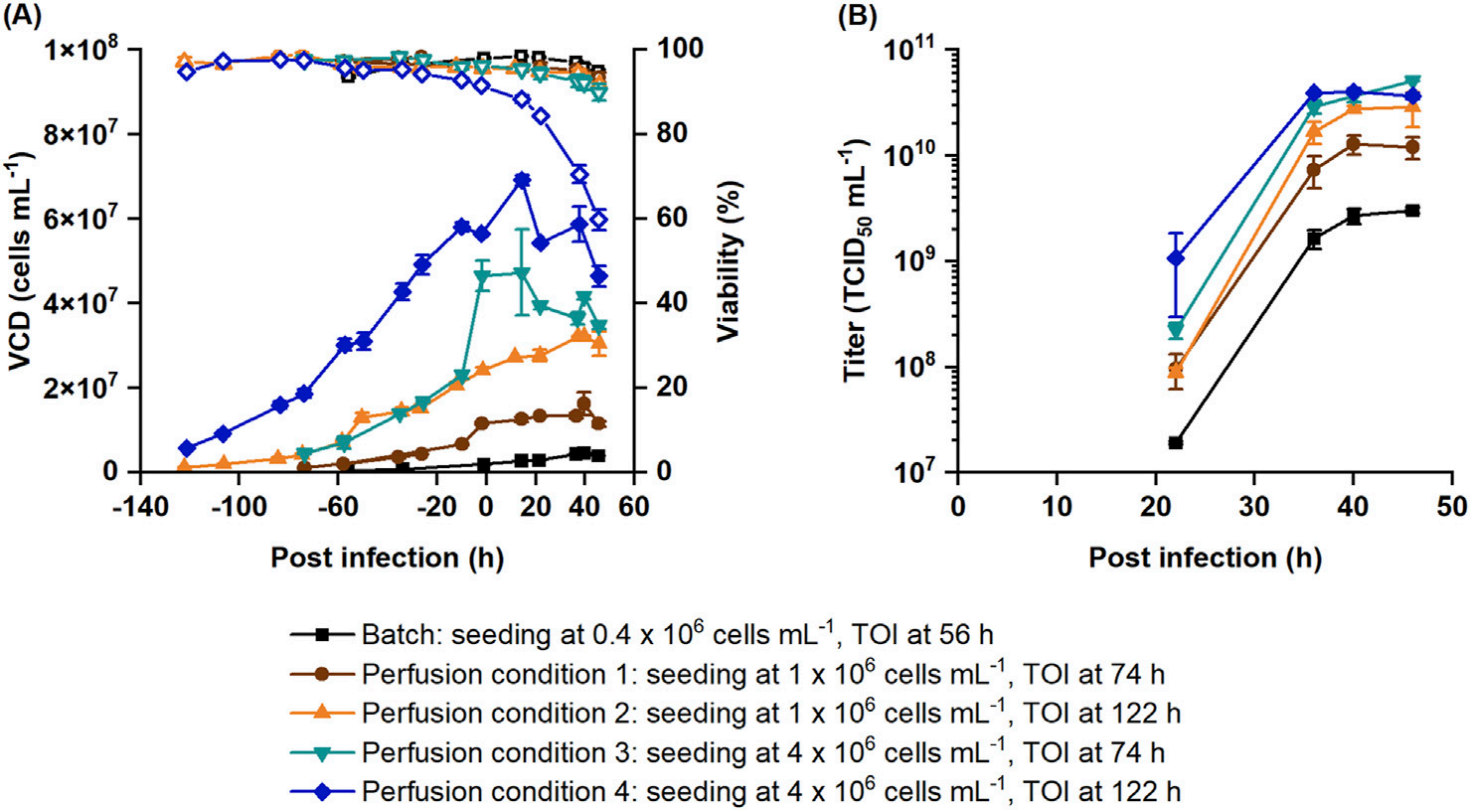
Optimized recombinant VSV-GP perfusion production processes in comparison to the batch production process. Cells were seeded at 1 × 10^6^ or 4 × 10^6^ cells mL^-1^ and infected at either 74 or 122 h. **(A)** VCD (filled symbols) and viability (empty symbols) obtained with Nucleocounter^®^ NC-202™ are shown along with the **(B)** infectious titer. Data represent the mean and standard deviation of biological duplicates.

The perfusion process was optimized to maximize the infectious virus titer at 46 hpi based on the fitted statistical model. Since the seeding cell density and the TOI both influence the medium consumption as well as the duration of the process, they also impact the P_V_. To assess this impact, four conditions were selected for optimization. Bioreactors were seeded at either 1 × 10^6^ or 4 × 10^6^ cells mL^-1^ and infected at either 74 or 122 h of cultivation. These optimized processes were compared to the batch production process in terms of titer, CSVY, STY and P_V_ (Table 3; Figure 4).

**TABLE 3:** Comparison of *batch and perfusion processes for recombinant VSV-GP production. Perfusion cultures were seeded* at 1 × 10^6^ or 4 × 10^6^ cells mL^-1^ and infected at either 74 or 122 h with an MOI of 0.0004. *Values are reported as the mean and standard deviation of biological duplicates.*

	Batch	Perfusion condition 1	Perfusion condition 2	Perfusion condition 3	Perfusion condition 4
Seeding VCD [x 10^6^ cells mL^-1^]	0.4	1.0	1.0	4.0	4.0
TOI [h]	56	74	122	74	122
log_10_ MOI	−3.4	−3.4	−3.4	−3.4	−3.4
CSPR after TOI [nL cell^-1^ d^-1^]	n.a	0.051	0.049	0.048	0.045
Perfusion pause after TOI [h]	n.a	1.3	1.1	1.1	1.3
Crossflow rate after TOI [mL min^-1^]	n.a	44	46	48	55
Max VCD [x 10^6^ cells mL^-1^]	4.6	16.2 ± 2.7	32.2 ± 0.1	47.3 ± 10.2	69.2 ± 1.2
Max titer [x 10^9^ TCID_50_ mL^-1^]	3.0 ± 0.2	12.8 ± 2.6	29.0 ± 10.6	51.1	40.2 ± 2.7
CSVY [TCID_50_ cell^-1^]	656 ± 51	790 ± 29	894 ± 320	989 ± 73	553 ± 12
STY [x 10^8^ TCID_50_ mL^-1^ d^-1^]	7.1 ± 0.5	27.1 ± 5.5	41.5 ± 15.1	102.0	59.7 ± 4.0
P_V_ [x 10^8^ TCID_50_ mL^-1^ d^-1^]	7.1 ± 0.5	11.3 ± 2.5	8.7 ± 3.3	21.1 ± 0.1	6.4 ± 0.3
Used medium [mL]	200	527 ± 10	1050 ± 17	1067 ± 3	2060 ± 49

Regardless of the seeding VCD or TOI, the settings of the optimized parameters of the perfusion cultivations were similar (Table 3). Using the lower MOI of 0.0004, the perfusion should start 1.1–1.3 h after the infection with a comparable CSPR as before the infection (0.045–0.051 nL cell^-1^ d^-1^). The optimal range for crossflow rate was 44–55 mL min^-1^.

Reference batch cultures were infected at 2.0 ± 0.1 × 10^6^ cells mL^-1^. Perfusion cultures reached VCDs between 11.6 ± 0.3 × 10^6^ and 56.5 ± 0.1 × 10^6^ cells mL^-1^ at the time of infection depending on the seeding VCD and time of infection. Batch and perfusion cultures with lower VCDs (condition 1 and 2) continued to grow after infection. Perfusion cultures with higher VCDs (condition 3 and 4) only showed minor cell growth post infection. The viability of perfusion cultures with the highest VCD (condition 4) rapidly decreased after infection to 59.8% ± 2.4% by 46 hpi. Presumably, this decrease in viability was triggered by the increased shear stress due to higher oxygen flow rates (0.29 ± 0.01 VVM) and bubble entrapment within the membrane (Figure 4A), which is consistent with earlier observations (Figure 1A).

While batch cultures had a titer of 3.0 ± 0.2 × 10^9^ TCID_50_ mL^-1^, perfusion cultures produced between 1.3 × 10^10^ and 5.1 × 10^10^ TCID_50_ mL^-1^, which is 4.3–16.9-fold higher than in batch cultures (Table 3; Figure 4B). These results closely match the prediction of the statistical model (Supplementary Figure S2), i.e., the results were within the 95% prediction intervals of the model. As described in Table 3, the CSVY improved with perfusion, with a maximum observed for condition 3. However, perfusion cultures with the highest cell density (condition 4) had slightly lower CSVYs than the batch cultures (Table 3), which is possibly related to the lower cell viabilities after infection (Figure 4A).

Increasing the seeding cell density from 1 × 10^6^ cells mL^-1^ to 4 × 10^6^ cells mL^-1^ also increased the STY up to 3.8-fold and the P_V_ by 1.9-fold. The highest STY of 1.0 × 10^10^ TCID_50_ mL^-1^ d^-1^ was achieved under perfusion condition 3, which is 14.4-fold higher than the STY with batch cultivation. Perfusion condition 3 also allowed to increase the P_V_ by 3.0-fold compared to the batch cultivation. Perfusion conditions 1, 2 and 4 had a comparable P_V_ to the batch cultures (Table 3).

In summary, perfusion cultivation allowed to infect cells up to 46.6 ± 3.6 × 10^6^ cells mL^-1^ and increased both infectious titer and STY without a decrease in the CSVY compared to the reference batch cultivation. Also, increasing the seeding VCD and infecting cells at an earlier timepoint was beneficial to improve both STY and P_V_.

## Discussion

This work aimed to increase the titer of the recombinant VSV-GP production process with suspension HEK293 cells by using TFF perfusion with virus retention. For this purpose, we initially investigated the growth of HEK293 cells with three different perfusion exchange rates. Subsequently, input parameters were screened and optimized for high infectious titer in Ambr^®^ 250 bioreactors.

Perfusion cultivation enabled to reach high viability cell cultures (≥90%) up to maximum 59.0 × 10^6^ cells mL^-1^, which is approximately 5-fold higher than in the classical batch cultivation process. Higher medium exchange rates slightly supported higher specific growth rates, reaching 0.022, 0.025 and 0.027 h^-1^ with perfusion rates of 0.029, 0.051 or 0.073 nL cell^-1^ d^-1^, respectively. These values are comparable to the previously reported specific growth rates of recombinant HEK293 cells obtained with ATF perfusion (up to approximately 0.6 d^-1^ or 0.025 h^-1^) (Schwarz et al., 2020) or perfusion with acoustic separator (0.03 h^-1^) (Henry et al., 2005). Once the cell cultures reached 56.1 × 10^6^–59.0 × 10^6^ cells mL^-1^, the viability gradually decreased while the cell density continued to increase. Schwarz et al. correlated the reduced viability of HEK293 cell cultures at high cell densities with the higher oxygen flow rate and stirring speed (Schwarz et al., 2020). While the stirring speed was constant in this study, the oxygen flow rate continuously increased to keep the DO at set point. Therefore, it is possible that high shear forces caused by bubble ruptures damaged the cells (Hu et al., 2011). In addition, it is well described that high shear stress should be avoided during virus production to minimize virus damage (Grein et al., 2019). Accordingly, cell cultures with viable cells densities above 59 × 10^6^ cells mL^-1^ were not considered for oncolytic VSV-GP production in this study.

To optimize the perfusion cultivation process, a DoE was conducted, and the VSV-GP production was modeled as a function of the studied parameters and their influence on the kinetics. The model revealed a complex relationship between the parameters and the titer of the perfusion process. Each input parameter had a significant impact on the titer. Additionally, several significant interactions were observed, reflecting the complexity of the biological system, where the effect of one factor changes as another factor is varied. The model exhibited a high *R* ^2^ value of 86.9%, indicating that the main sources of variance were identified. However, a portion of the variance remains unexplained, which might suggest even more complex kinetics in the perfusion process. Further investigation into these effects and the kinetics could provide additional insights.

The concentration of viable cells as available substrate for the virus production has a substantial impact on the attainable titer. Therefore, increasing the seeding cell density was beneficial to increase the infectious titer. Similarly, choosing a later timepoint for infection also improved the titer.

Besides the improved titer, increasing the seeding cell density from 1 × 10^6^ cells mL^-1^ to 4 × 10^6^ cells mL^-1^ also increased the STY up to 3.8-fold and the P_V_ up to 1.9-fold. To meet the high cell demand for seeding bioreactors at higher cell densities, the volume of N-1 stage cultures had to be increased. Additionally, N-1 stage cultures had to be concentrated by centrifugation to ensure that a maximum split ratio of 20% is not exceeded in the N-stage bioreactor. The implementation of this strategy is challenging in large-scale production. Several authors have instead proposed N-1 perfusion to enable higher seeding cell densities for the N-stage fed-batch process. The goal has been to decrease the process time (Yang et al., 2014) or increase the titer (Stepper et al., 2020). Due to the high cell density of N-1 perfusion cultures, the number and size of the vessels in the seed train can also be reduced, which shortens the total cultivation time of the seed train (Stepper et al., 2020).

The optimal crossflow rate of the virus production process lay in the range between 44 and 55 mL min^-1^, which corresponds to a maximum shear rate of approximately 950–1150 s^-1^ in the perfusion loop as represented in the tubing and fittings. By increasing the crossflow rate, the shear rate also increased (1500 s^-1^ with 70 mL min^-1^ crossflow rate) and reduced the infectious virus titer. Zhang et al. recommended the threshold of 1266 s^-1^ shear rate to avoid shear-induced cell damage in hybridoma cell cultures during perfusion (Zhang et al., 1993). This threshold was exceeded in cultures with crossflow rate above 60 mL min^-1^. In another study, however, no considerable decrease in the cell growth has been observed at 2482 s^-1^ shear rate for HEK293 cells (Zhan et al., 2020). Hydrodynamic shear stress can also damage viruses (Loewe et al., 2019). Interestingly, crossflow rates below the optimal range (44–55 mL min^-1^) also led to a slight decrease in titer. This decrease may be attributed to non-specific virus adsorption to the mPES membrane. Additionally, applying a lower crossflow rate increases the residence time in the perfusion loop, which represents a non-controlled environment and might negatively impact the process (Chotteau, 2014). Further studies are necessary to understand the impact of residence time in more depth.

A short perfusion pause after infection is often used in virus production processes to minimize the virus washout in the external perfusion filter module. This practice is particularly important for processes with low MOIs to ensure an efficient virus infection (Yuk et al., 2004; Gálvez et al., 2012; Genzel et al., 2014). Although the filter cut-off in this study was smaller than the virus, pausing the perfusion after infection may prevent non-specific adsorption of the virus to the membrane, which could reduce the effective MOI. Our study indicates that a perfusion pause of 1.1–1.3 h is optimal for supporting VSV-GP entry into cells. Longer perfusion pauses were found to hinder the virus production. In an adenoviral vector production process, the decreased virus productivity was linked to the reduced metabolic activity of HEK293 cells (Henry et al., 2004). A following study found that HEK293 cells exhibited a lower ATP production rate and virus yield when subjected to low perfusion rates at high cell density (Henry et al., 2005). Therefore, it is possible that the metabolic activity of HEK293 cells also decreased during a longer perfusion pause, leading to a reduced virus titer. Furthermore, we also observed lower titers at lower medium exchange rates. Applying a higher MOI can counterbalance the impact of lower perfusion rate and decrease medium consumption. Alternatively, to spare on the expensive virus seed stock, a higher perfusion rate and a lower MOI can be employed.

Only a few perfusion cultivation processes have been reported for VSV production so far. Paillet et al. showed the feasibility to use suspension Vero cell cultures for VSV production in perfusion (Paillet et al., 2009). Recently, a perfusion process using tangential flow depth filtration (TFDF) was reported for producing VSV-based vectors with suspension BHK-21 and HEK293-SF cells with continuous virus harvest. In the HEK293-SF cell-based process, cells reached a maximum concentration of 11.3 × 10^6^ cells mL^-1^ and exhibited 1.9 and 1.1-fold higher STY compared to the batch culture (Göbel et al., 2024). Our work additionally shows the feasibility of using TFF perfusion with virus retention to increase the viral output of the oncolytic VSV-GP production process. Furthermore, the optimized TFF process allowed to infect cultures at a wide range of cell density (up to 46.6 × 10^6^ cells mL^-1^) without a decrease in CSVY. The optimized process exhibited up to 14.4-fold higher STY compared to the batch cultivation.

## Conclusion

This work has improved the oncolytic VSV-GP production process with suspension HEK293 cells by using TFF perfusion. The optimized TFF process allowed to achieve up to 16.9-fold higher titer (5.1 × 10^10^ TCID_50_ mL^-1^), up to 14.4-fold higher STY and up to 3.0-fold increase in the P_V_ compared to the batch cultivation. Due to the higher titers and higher levels of impurities (such as cell debris, host cell DNA, host cell protein) in high cell density culture harvests, modifications to the reference downstream process, based on batch cultivation, might become necessary. These modifications may involve adding a centrifugation step before depth filtration and optimizing the depth filtration and chromatography steps to handle higher loads and impurities. Overall, this work presents the potential of the TFF perfusion process for efficiently producing oncolytic VSV-GP.

## Data Availability

The original contributions presented in the study are included in the article/Supplementary Material, further inquiries can be directed to the corresponding authors.

## Abbreviations

AICc, Akaike information criterion corrected for small sample size; ANOVA, analysis of variance; ATF, alternating tangential flow filtration; BHK-21, baby hamster kidney 21 cells; CV, coefficient of variation; CSPR, cell-specific perfusion rate; DFFITs, difference in fits; DO, dissolved oxygen; DoE, design of experiments; G, glycoprotein of VSV; GP, glycoprotein of LCMV; HEK293, human embryonic kidney 293 cells; hpi, hours post infection; LCMV, lymphocytic choriomeningitis virus; MOI, multiplicity of infection; mPES, modified polyether sulfone; OLS, ordinary least square; PV, volumetric productivity; REML, restricted maximum likelihood analysis; RV, reactor volume; STY, space-time yield; TCID50, tissue culture infectious dose 50 assay; TFDF, tangential flow depth filtration; TFF, tangential flow filtration; TOI, time of infection; VCD, viable cell density; VSV, vesicular stomatitis virus; VSV-GP, vesicular stomatitis virus variant that was pseudotyped with LCMV-GP; VVM, vessel volumes per minute.
