# Supplementary material for: Perfusion process with tangential flow filtration for oncolytic VSV-GP production
Source: Front Bioeng Biotechnol. 2025 May 30;13:1588293. doi: 10.3389/fbioe.2025.1588293 (PMC12162514; doi:10.3389/fbioe.2025.1588293)
Supplement: Supplementary file 1 [file DataSheet1.docx]

Supplementary Material

**Supplementary Table S1. Input parameter settings and measured titers of 45 bioreactor (BR) runs. Experiments were conducted in four blocks by varying the seeding VCD, TOI, MOI as well as the CSPR, duration of perfusion pause and crossflow rate after TOI. Virus samples were collected at 22, 36, 40 and 46 hpi.**

| **Block** | **BR  No.** | **Seeding VCD [x 10^6^ cells mL^-1^]** | **TOI [h]** | **log_10_ MOI** | **CSPR after TOI [nL cell^-1^ d^-1^]** | **Perfusion  pause after  TOI [h]** | **Crossflow rate  after TOI  [mL min^-1^]** | **hpi** | **Titer [TCID_50_ mL^-1^]** |
| --- | --- | --- | --- | --- | --- | --- | --- | --- | --- |
| 1 | 1 | 4 | 98 | -2.4 | 0.033 | 8 | 40 | 22 | 4.87E+07 |
| 1 | 1 | 4 | 98 | -2.4 | 0.033 | 8 | 40 | 36 | 2.37E+09 |
| 1 | 1 | 4 | 98 | -2.4 | 0.033 | 8 | 40 | 40 | 1.62E+10 |
| 1 | 1 | 4 | 98 | -2.4 | 0.033 | 8 | 40 | 46 | 2.49E+10 |
| 1 | 2 | 4 | 74 | -3.4 | 0.015 | 16 | 25 | 22 | 8.66E+05 |
| 1 | 2 | 4 | 74 | -3.4 | 0.015 | 16 | 25 | 36 | 4.87E+07 |
| 1 | 2 | 4 | 74 | -3.4 | 0.015 | 16 | 25 | 40 | 1.10E+08 |
| 1 | 2 | 4 | 74 | -3.4 | 0.015 | 16 | 25 | 46 | 7.15E+08 |
| 1 | 3 | 4 | 74 | -1.4 | 0.051 | 16 | 25 | 22 | 1.38E+08 |
| 1 | 3 | 4 | 74 | -1.4 | 0.051 | 16 | 25 | 36 | 1.20E+10 |
| 1 | 3 | 4 | 74 | -1.4 | 0.051 | 16 | 25 | 40 | 8.66E+09 |
| 1 | 3 | 4 | 74 | -1.4 | 0.051 | 16 | 25 | 46 | 2.26E+10 |
| 1 | 4 | 1 | 74 | -1.4 | 0.015 | 16 | 25 | 22 | 1.27E+09 |
| 1 | 4 | 1 | 74 | -1.4 | 0.015 | 16 | 25 | 36 | 8.25E+09 |
| 1 | 4 | 1 | 74 | -1.4 | 0.015 | 16 | 25 | 40 | 6.19E+09 |
| 1 | 4 | 1 | 74 | -1.4 | 0.015 | 16 | 25 | 46 | 6.19E+09 |
| 1 | 5 | 2.5 | 122 | -2.4 | 0.051 | 16 | 70 | 22 | 1.33E+07 |
| 1 | 5 | 2.5 | 122 | -2.4 | 0.051 | 16 | 70 | 36 | 2.61E+09 |
| 1 | 5 | 2.5 | 122 | -2.4 | 0.051 | 16 | 70 | 40 | 7.87E+09 |
| 1 | 5 | 2.5 | 122 | -2.4 | 0.051 | 16 | 70 | 46 | 1.78E+10 |
| 1 | 6 | 1 | 122 | -3.4 | 0.015 | 16 | 70 | 22 | 4.42E+05 |
| 1 | 6 | 1 | 122 | -3.4 | 0.015 | 16 | 70 | 36 | 3.32E+07 |
| 1 | 6 | 1 | 122 | -3.4 | 0.015 | 16 | 70 | 40 | 2.05E+08 |
| 1 | 6 | 1 | 122 | -3.4 | 0.015 | 16 | 70 | 46 | 1.47E+09 |
| 1 | 7 | 1 | 74 | -1.4 | 0.051 | 1 | 70 | 22 | 2.15E+09 |
| 1 | 7 | 1 | 74 | -1.4 | 0.051 | 1 | 70 | 36 | 5.11E+09 |
| 1 | 7 | 1 | 74 | -1.4 | 0.051 | 1 | 70 | 40 | 5.62E+09 |
| 1 | 7 | 1 | 74 | -1.4 | 0.051 | 1 | 70 | 46 | 5.36E+09 |
| 1 | 8 | 2.5 | 122 | -3.4 | 0.033 | 1 | 40 | 22 | 2.84E+07 |
| 1 | 8 | 2.5 | 122 | -3.4 | 0.033 | 1 | 40 | 36 | 5.90E+09 |
| 1 | 8 | 2.5 | 122 | -3.4 | 0.033 | 1 | 40 | 40 | 1.78E+10 |
| 1 | 8 | 2.5 | 122 | -3.4 | 0.033 | 1 | 40 | 46 | 4.87E+10 |
| 1 | 9 | 1 | 98 | -3.4 | 0.015 | 1 | 25 | 22 | 8.66E+06 |
| 1 | 9 | 1 | 98 | -3.4 | 0.015 | 1 | 25 | 36 | 1.29E+09 |
| 1 | 9 | 1 | 98 | -3.4 | 0.015 | 1 | 25 | 40 | 3.01E+09 |
| 1 | 9 | 1 | 98 | -3.4 | 0.015 | 1 | 25 | 46 | 1.10E+10 |
| 1 | 10 | 2.5 | 98 | -1.4 | 0.015 | 8 | 70 | 22 | 1.69E+08 |
| 1 | 10 | 2.5 | 98 | -1.4 | 0.015 | 8 | 70 | 36 | 8.25E+09 |
| 1 | 10 | 2.5 | 98 | -1.4 | 0.015 | 8 | 70 | 40 | 1.27E+10 |
| 1 | 10 | 2.5 | 98 | -1.4 | 0.015 | 8 | 70 | 46 | 1.69E+10 |
| 1 | 11 | 1 | 74 | -3.4 | 0.051 | 16 | 40 | 22 | 1.54E+07 |
| 1 | 11 | 1 | 74 | -3.4 | 0.051 | 16 | 40 | 36 | 2.87E+09 |
| 1 | 11 | 1 | 74 | -3.4 | 0.051 | 16 | 40 | 40 | 7.29E+09 |
| 1 | 11 | 1 | 74 | -3.4 | 0.051 | 16 | 40 | 46 | 6.49E+09 |
| 1 | 12 | 1 | 122 | -1.4 | 0.033 | 1 | 40 | 22 | 5.62E+08 |
| 1 | 12 | 1 | 122 | -1.4 | 0.033 | 1 | 40 | 36 | 1.87E+10 |
| 1 | 12 | 1 | 122 | -1.4 | 0.033 | 1 | 40 | 40 | 2.15E+10 |
| 1 | 12 | 1 | 122 | -1.4 | 0.033 | 1 | 40 | 46 | 3.01E+10 |
| 2 | 13 | 4 | 74 | -3.4 | 0.051 | 1 | 25 | 22 | 7.50E+07 |
| 2 | 13 | 4 | 74 | -3.4 | 0.051 | 1 | 25 | 36 | 1.47E+10 |
| 2 | 13 | 4 | 74 | -3.4 | 0.051 | 1 | 25 | 40 | 2.74E+10 |
| 2 | 13 | 4 | 74 | -3.4 | 0.051 | 1 | 25 | 46 | 3.16E+10 |
| 2 | 14 | 2.5 | 98 | -2.4 | 0.033 | 16 | 25 | 22 | 1.78E+07 |
| 2 | 14 | 2.5 | 98 | -2.4 | 0.033 | 16 | 25 | 36 | 1.69E+09 |
| 2 | 14 | 2.5 | 98 | -2.4 | 0.033 | 16 | 25 | 40 | 7.87E+09 |
| 2 | 14 | 2.5 | 98 | -2.4 | 0.033 | 16 | 25 | 46 | 2.26E+10 |
| 2 | 15 | 2.5 | 98 | -3.4 | 0.051 | 8 | 70 | 22 | 1.05E+07 |
| 2 | 15 | 2.5 | 98 | -3.4 | 0.051 | 8 | 70 | 36 | 3.01E+09 |
| 2 | 15 | 2.5 | 98 | -3.4 | 0.051 | 8 | 70 | 40 | 1.15E+10 |
| 2 | 15 | 2.5 | 98 | -3.4 | 0.051 | 8 | 70 | 46 | 2.61E+10 |
| 2 | 16 | 1 | 74 | -3.4 | 0.015 | 8 | 70 | 22 | 5.90E+07 |
| 2 | 16 | 1 | 74 | -3.4 | 0.015 | 8 | 70 | 36 | 4.64E+09 |
| 2 | 16 | 1 | 74 | -3.4 | 0.015 | 8 | 70 | 40 | 1.15E+10 |
| 2 | 16 | 1 | 74 | -3.4 | 0.015 | 8 | 70 | 46 | 1.21E+10 |
| 2 | 17 | 4 | 122 | -3.4 | 0.015 | 8 | 70 | 22 | 1.47E+07 |
| 2 | 17 | 4 | 122 | -3.4 | 0.015 | 8 | 70 | 36 | 1.69E+09 |
| 2 | 17 | 4 | 122 | -3.4 | 0.015 | 8 | 70 | 40 | 5.62E+09 |
| 2 | 17 | 4 | 122 | -3.4 | 0.015 | 8 | 70 | 46 | 2.87E+10 |
| 2 | 18 | 1 | 122 | -2.4 | 0.015 | 8 | 40 | 22 | 3.65E+07 |
| 2 | 18 | 1 | 122 | -2.4 | 0.015 | 8 | 40 | 36 | 2.05E+09 |
| 2 | 18 | 1 | 122 | -2.4 | 0.015 | 8 | 40 | 40 | 4.87E+09 |
| 2 | 18 | 1 | 122 | -2.4 | 0.015 | 8 | 40 | 46 | 2.15E+10 |
| 2 | 19 | 1 | 122 | -3.4 | 0.051 | 8 | 25 | 22 | 4.22E+06 |
| 2 | 19 | 1 | 122 | -3.4 | 0.051 | 8 | 25 | 36 | 7.87E+08 |
| 2 | 19 | 1 | 122 | -3.4 | 0.051 | 8 | 25 | 40 | 2.87E+09 |
| 2 | 19 | 1 | 122 | -3.4 | 0.051 | 8 | 25 | 46 | 1.54E+10 |
| 2 | 20 | 4 | 74 | -1.4 | 0.015 | 1 | 40 | 22 | 1.33E+09 |
| 2 | 20 | 4 | 74 | -1.4 | 0.015 | 1 | 40 | 36 | 1.96E+10 |
| 2 | 20 | 4 | 74 | -1.4 | 0.015 | 1 | 40 | 40 | 2.05E+10 |
| 2 | 20 | 4 | 74 | -1.4 | 0.015 | 1 | 40 | 46 | 3.83E+10 |
| 2 | 21 | 4 | 122 | -1.4 | 0.051 | 1 | 70 | 22 | 1.54E+10 |
| 2 | 21 | 4 | 122 | -1.4 | 0.051 | 1 | 70 | 36 | 2.49E+10 |
| 2 | 21 | 4 | 122 | -1.4 | 0.051 | 1 | 70 | 40 | 3.16E+10 |
| 2 | 21 | 4 | 122 | -1.4 | 0.051 | 1 | 70 | 46 | 3.01E+10 |
| 2 | 22 | 4 | 122 | -3.4 | 0.051 | 16 | 40 | 22 | 5.62E+06 |
| 2 | 22 | 4 | 122 | -3.4 | 0.051 | 16 | 40 | 36 | 2.37E+09 |
| 2 | 22 | 4 | 122 | -3.4 | 0.051 | 16 | 40 | 40 | 7.50E+09 |
| 2 | 22 | 4 | 122 | -3.4 | 0.051 | 16 | 40 | 46 | 2.61E+10 |
| 2 | 23 | 4 | 122 | -1.4 | 0.015 | 16 | 40 | 22 | 8.25E+08 |
| 2 | 23 | 4 | 122 | -1.4 | 0.015 | 16 | 40 | 36 | 2.05E+10 |
| 2 | 23 | 4 | 122 | -1.4 | 0.015 | 16 | 40 | 40 | 3.48E+10 |
| 2 | 23 | 4 | 122 | -1.4 | 0.015 | 16 | 40 | 46 | 2.94E+10 |
| 2 | 24 | 4 | 74 | -2.4 | 0.033 | 16 | 70 | 22 | 2.37E+07 |
| 2 | 24 | 4 | 74 | -2.4 | 0.033 | 16 | 70 | 36 | 3.48E+09 |
| 2 | 24 | 4 | 74 | -2.4 | 0.033 | 16 | 70 | 40 | 1.33E+10 |
| 2 | 24 | 4 | 74 | -2.4 | 0.033 | 16 | 70 | 46 | 2.74E+10 |
| 3 | 25 | 1 | 98 | -1.4 | 0.051 | 16 | 70 | 22 | 1.65E+09 |
| 3 | 25 | 1 | 98 | -1.4 | 0.051 | 16 | 70 | 36 | 1.40E+10 |
| 3 | 25 | 1 | 98 | -1.4 | 0.051 | 16 | 70 | 40 | 1.21E+10 |
| 3 | 25 | 1 | 98 | -1.4 | 0.051 | 16 | 70 | 46 | 1.05E+10 |
| 3 | 26 | 4 | 122 | -1.4 | 0.015 | 1 | 25 | 22 | 9.53E+09 |
| 3 | 26 | 4 | 122 | -1.4 | 0.015 | 1 | 25 | 36 | 3.01E+10 |
| 3 | 26 | 4 | 122 | -1.4 | 0.015 | 1 | 25 | 40 | 3.01E+10 |
| 3 | 26 | 4 | 122 | -1.4 | 0.015 | 1 | 25 | 46 | 3.16E+10 |
| 3 | 27 | 1 | 122 | -2.4 | 0.033 | 1 | 70 | 22 | 4.02E+08 |
| 3 | 27 | 1 | 122 | -2.4 | 0.033 | 1 | 70 | 36 | 1.87E+10 |
| 3 | 27 | 1 | 122 | -2.4 | 0.033 | 1 | 70 | 40 | 3.65E+10 |
| 3 | 27 | 1 | 122 | -2.4 | 0.033 | 1 | 70 | 46 | 3.65E+10 |
| 3 | 28 | 4 | 74 | -3.4 | 0.033 | 1 | 70 | 22 | 6.19E+07 |
| 3 | 28 | 4 | 74 | -3.4 | 0.033 | 1 | 70 | 36 | 1.00E+10 |
| 3 | 28 | 4 | 74 | -3.4 | 0.033 | 1 | 70 | 40 | 1.78E+10 |
| 3 | 28 | 4 | 74 | -3.4 | 0.033 | 1 | 70 | 46 | 2.61E+10 |
| 3 | 29 | 2.5 | 74 | -1.4 | 0.033 | 8 | 40 | 22 | 3.48E+09 |
| 3 | 29 | 2.5 | 74 | -1.4 | 0.033 | 8 | 40 | 36 | 1.62E+10 |
| 3 | 29 | 2.5 | 74 | -1.4 | 0.033 | 8 | 40 | 40 | 1.05E+10 |
| 3 | 29 | 2.5 | 74 | -1.4 | 0.033 | 8 | 40 | 46 | 1.33E+10 |
| 3 | 30 | 2.5 | 98 | -2.4 | 0.051 | 1 | 40 | 22 | 1.54E+09 |
| 3 | 30 | 2.5 | 98 | -2.4 | 0.051 | 1 | 40 | 36 | 1.62E+10 |
| 3 | 30 | 2.5 | 98 | -2.4 | 0.051 | 1 | 40 | 40 | 2.15E+10 |
| 3 | 30 | 2.5 | 98 | -2.4 | 0.051 | 1 | 40 | 46 | 3.01E+10 |
| 3 | 31 | 1 | 122 | -1.2 | 0.051 | 1 | 70 | 22 | 8.25E+09 |
| 3 | 31 | 1 | 122 | -1.2 | 0.051 | 1 | 70 | 36 | 1.62E+10 |
| 3 | 31 | 1 | 122 | -1.2 | 0.051 | 1 | 70 | 40 | 2.61E+10 |
| 3 | 31 | 1 | 122 | -1.2 | 0.051 | 1 | 70 | 46 | 2.87E+10 |
| 3 | 32 | 1 | 122 | -2.2 | 0.051 | 1 | 70 | 22 | 1.62E+09 |
| 3 | 32 | 1 | 122 | -2.2 | 0.051 | 1 | 70 | 36 | 1.78E+10 |
| 3 | 32 | 1 | 122 | -2.2 | 0.051 | 1 | 70 | 40 | 1.21E+10 |
| 3 | 32 | 1 | 122 | -2.2 | 0.051 | 1 | 70 | 46 | 3.40E+10 |
| 3 | 33 | 1 | 122 | -3.2 | 0.051 | 1 | 70 | 22 | 1.33E+08 |
| 3 | 33 | 1 | 122 | -3.2 | 0.051 | 1 | 70 | 36 | 1.15E+10 |
| 3 | 33 | 1 | 122 | -3.2 | 0.051 | 1 | 70 | 40 | 2.15E+10 |
| 3 | 33 | 1 | 122 | -3.2 | 0.051 | 1 | 70 | 46 | 3.01E+10 |
| 4 | 34 | 1.4 | 122 | -3.4 | 0.049 | 1.4 | 53 | 22 | 4.22E+08 |
| 4 | 34 | 1.4 | 122 | -3.4 | 0.049 | 1.4 | 53 | 36 | 2.61E+10 |
| 4 | 34 | 1.4 | 122 | -3.4 | 0.049 | 1.4 | 53 | 40 | 3.83E+10 |
| 4 | 34 | 1.4 | 122 | -3.4 | 0.049 | 1.4 | 53 | 46 | 5.62E+10 |
| 4 | 35 | 1.4 | 122 | -3.4 | 0.049 | 1.4 | 53 | 22 | 2.94E+08 |
| 4 | 35 | 1.4 | 122 | -3.4 | 0.049 | 1.4 | 53 | 36 | 3.48E+10 |
| 4 | 35 | 1.4 | 122 | -3.4 | 0.049 | 1.4 | 53 | 40 | 3.65E+10 |
| 4 | 35 | 1.4 | 122 | -3.4 | 0.049 | 1.4 | 53 | 46 | 2.15E+10 |
| 4 | 36 | 3.7 | 122 | -3.3 | 0.046 | 1 | 55 | 22 | 9.53E+08 |
| 4 | 36 | 3.7 | 122 | -3.3 | 0.046 | 1 | 55 | 36 | 3.48E+10 |
| 4 | 36 | 3.7 | 122 | -3.3 | 0.046 | 1 | 55 | 40 | 4.87E+10 |
| 4 | 36 | 3.7 | 122 | -3.3 | 0.046 | 1 | 55 | 46 | 3.48E+10 |
| 4 | 37 | 3.7 | 122 | -3.3 | 0.046 | 1 | 55 | 22 | 1.15E+09 |
| 4 | 37 | 3.7 | 122 | -3.3 | 0.046 | 1 | 55 | 36 | 3.83E+10 |
| 4 | 37 | 3.7 | 122 | -3.3 | 0.046 | 1 | 55 | 40 | 3.32E+10 |
| 4 | 37 | 3.7 | 122 | -3.3 | 0.046 | 1 | 55 | 46 | 3.01E+10 |
| 4 | 38 | 4.3 | 122 | -3.3 | 0.046 | 1.1 | 51 | 22 | 1.62E+09 |
| 4 | 38 | 4.3 | 122 | -3.3 | 0.046 | 1.1 | 51 | 36 | 4.02E+10 |
| 4 | 38 | 4.3 | 122 | -3.3 | 0.046 | 1.1 | 51 | 40 | 4.22E+10 |
| 4 | 38 | 4.3 | 122 | -3.3 | 0.046 | 1.1 | 51 | 46 | 3.83E+10 |
| 4 | 39 | 4.3 | 122 | -3.3 | 0.046 | 1.1 | 51 | 22 | 5.23E+08 |
| 4 | 39 | 4.3 | 122 | -3.3 | 0.046 | 1.1 | 51 | 36 | 3.83E+10 |
| 4 | 39 | 4.3 | 122 | -3.3 | 0.046 | 1.1 | 51 | 40 | 3.83E+10 |
| 4 | 39 | 4.3 | 122 | -3.3 | 0.046 | 1.1 | 51 | 46 | 3.48E+10 |
| 4 | 40 | 1.4 | 122 | -3.4 | 0.015 | 16 | 25 | 22 | 3.48E+06 |
| 4 | 40 | 1.4 | 122 | -3.4 | 0.015 | 16 | 25 | 36 | 3.83E+08 |
| 4 | 40 | 1.4 | 122 | -3.4 | 0.015 | 16 | 25 | 40 | 7.15E+08 |
| 4 | 40 | 1.4 | 122 | -3.4 | 0.015 | 16 | 25 | 46 | 7.15E+09 |
| 4 | 41 | 1.4 | 122 | -3.4 | 0.015 | 16 | 25 | 22 | 4.64E+06 |
| 4 | 41 | 1.4 | 122 | -3.4 | 0.015 | 16 | 25 | 36 | 4.02E+08 |
| 4 | 41 | 1.4 | 122 | -3.4 | 0.015 | 16 | 25 | 40 | 7.15E+08 |
| 4 | 41 | 1.4 | 122 | -3.4 | 0.015 | 16 | 25 | 46 | 5.90E+09 |
| 4 | 42 | 3.7 | 122 | -3.4 | 0.015 | 16 | 25 | 22 | 1.15E+07 |
| 4 | 42 | 3.7 | 122 | -3.4 | 0.015 | 16 | 25 | 36 | 1.54E+09 |
| 4 | 42 | 3.7 | 122 | -3.4 | 0.015 | 16 | 25 | 40 | 9.09E+09 |
| 4 | 42 | 3.7 | 122 | -3.4 | 0.015 | 16 | 25 | 46 | 1.65E+10 |
| 4 | 43 | 3.7 | 122 | -3.4 | 0.015 | 16 | 25 | 22 | 6.81E+06 |
| 4 | 43 | 3.7 | 122 | -3.4 | 0.015 | 16 | 25 | 36 | 8.25E+08 |
| 4 | 43 | 3.7 | 122 | -3.4 | 0.015 | 16 | 25 | 40 | 6.49E+09 |
| 4 | 43 | 3.7 | 122 | -3.4 | 0.015 | 16 | 25 | 46 | 1.54E+10 |
| 4 | 44 | 4.3 | 122 | -3.4 | 0.015 | 16 | 25 | 22 | 9.53E+06 |
| 4 | 44 | 4.3 | 122 | -3.4 | 0.015 | 16 | 25 | 36 | 1.33E+09 |
| 4 | 44 | 4.3 | 122 | -3.4 | 0.015 | 16 | 25 | 40 | 6.81E+09 |
| 4 | 44 | 4.3 | 122 | -3.4 | 0.015 | 16 | 25 | 46 | 5.36E+10 |
| 4 | 45 | 4.3 | 122 | -3.4 | 0.015 | 16 | 25 | 22 | 1.00E+07 |
| 4 | 45 | 4.3 | 122 | -3.4 | 0.015 | 16 | 25 | 36 | 5.90E+08 |
| 4 | 45 | 4.3 | 122 | -3.4 | 0.015 | 16 | 25 | 40 | 4.22E+09 |
| 4 | 45 | 4.3 | 122 | -3.4 | 0.015 | 16 | 25 | 46 | 3.32E+10 |

**
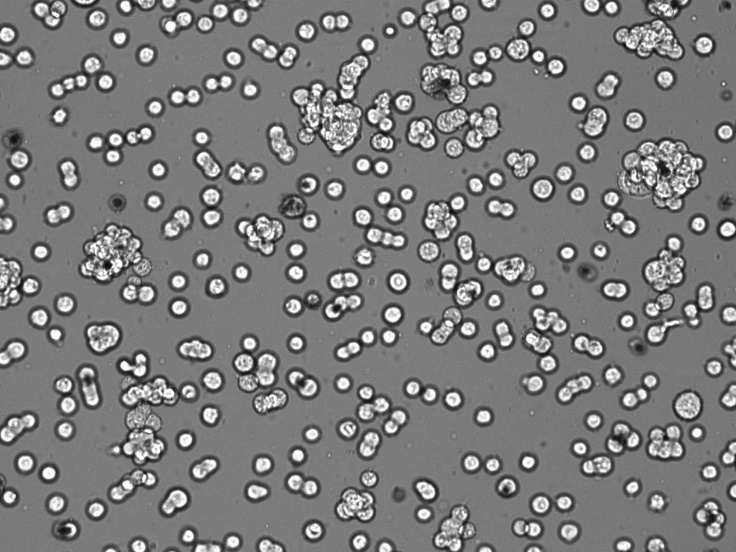
**

**Supplementary Figure S1. Aggregation of uninfected HEK293 cells during perfusion cultivation. Cells were stained with trypan blue before measurement with the BioProfile^®^ FLEX2 analyzer.**


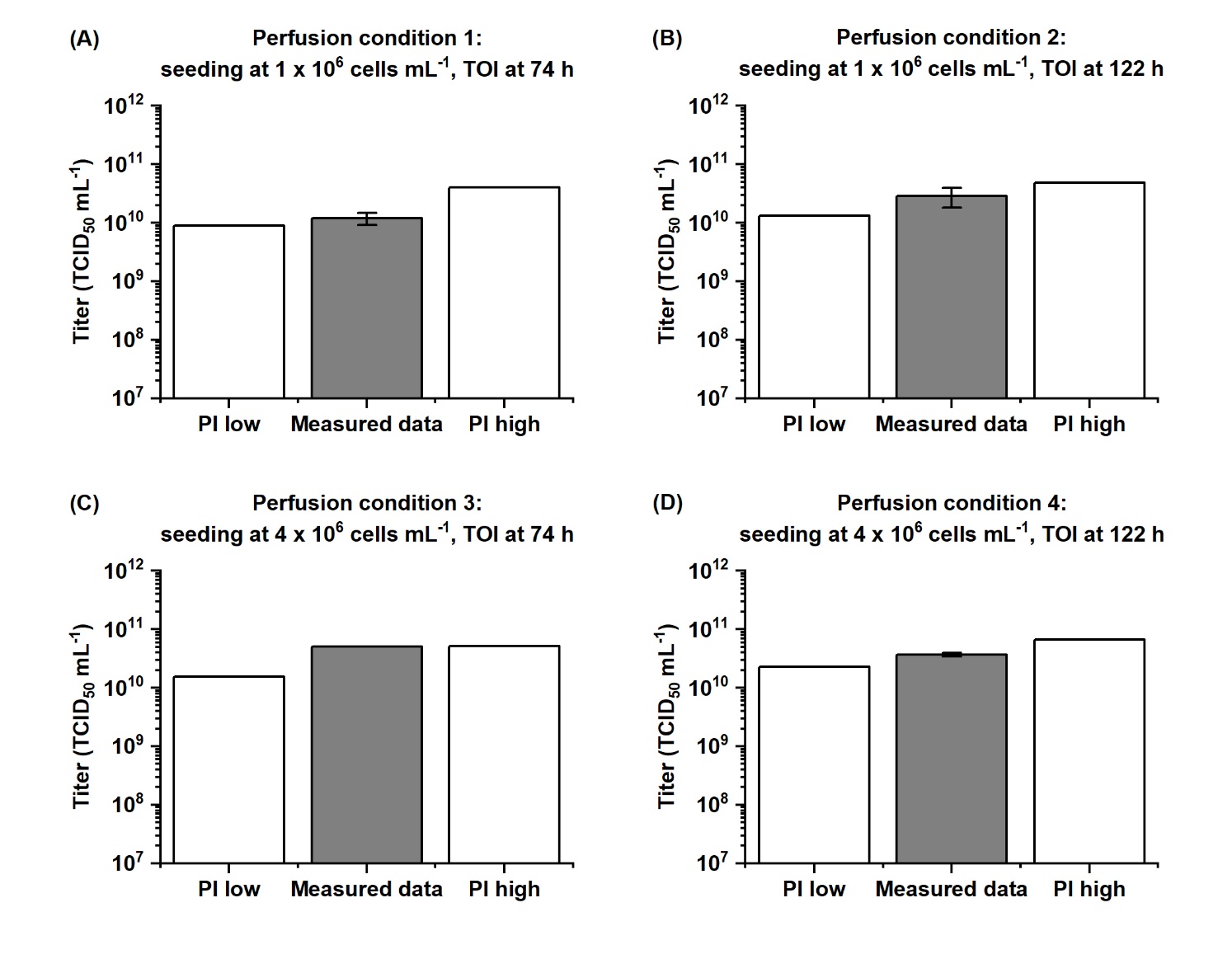


**Supplementary Figure S2. Confirmation of the DoE model for recombinant VSV-GP production with four perfusion conditions. Perfusion processes were optimized to maximize the infectious titer at 46 hpi by fixing the seeding VCD and TOI. Cells were seeded at 1 x 10^6^ or 4 x 10^6^ cells mL^-1^ and infected at either 74 h or 122 h. (A-D) Measured titers are represented as the mean and standard deviation of biological duplicates. The low and high limit of the prediction interval (PI) of the model is shown with 95% confidence.**
